# Adaptation to different communicative contexts: an eye tracking study of autistic adults

**DOI:** 10.1186/s11689-019-9265-1

**Published:** 2019-04-13

**Authors:** Julia Parish-Morris, Ashley A. Pallathra, Emily Ferguson, Brenna B. Maddox, Alison Pomykacz, Leat S. Perez, Leila Bateman, Juhi Pandey, Robert T. Schultz, Edward S. Brodkin

**Affiliations:** 1 Center for Autism Research, Children’s Hospital of Philadelphia, 5th Floor 2716 South Street, Philadelphia, PA 19146 USA; 2 Department of Psychiatry, Perelman School of Medicine at the University of Pennsylvania, Philadelphia, PA 19104 USA; 3 Department of Psychology, Catholic University of America, Washington, DC 20064 USA; 4 Department of Pediatrics, Perelman School of Medicine at the University of Pennsylvania, Philadelphia, PA 19104 USA

**Keywords:** Autism spectrum disorder, Adults, Eye gaze, Face processing/perception

## Abstract

**Background:**

Learning through social observation (i.e., watching other people interact) lays the foundation for later social skills and social cognition. However, social situations are often complex, and humans are only capable of attending to one aspect of a scene at a time. How do people choose where to allocate their visual resources when viewing complex social scenarios? For typically developing (TD) individuals, faces are often given priority. Depending upon context, however, it may be more useful to attend to other aspects of the environment, such as hands, tools, or background objects. Previous studies reported reduced face looking in individuals with autism spectrum disorder (ASD), but modulation of visual attention in response to contextual differences (e.g., according to *social richness*, or the presence/absence of communicative behaviors between two people) has only briefly been explored. In this study, we used eye-tracking technology to test the extent to which ASD adults and TD adults use social context to guide their gaze behavior.

**Methods:**

Fifty-five adults participated (28 with ASD). The location and duration of participants’ gaze were recorded while they watched a series of naturalistic social videos. Half of the videos depicted two people engaging in non-verbal communication (*rich* social scenes) while playing with toys. The other half depicted two people playing with toys separately, not interacting with each other (*lean* social scenes).

**Results:**

ASD and TD adults both increased their attention to faces in communicative contexts (*rich* social scenes) as compared to non-communicative contexts (*lean* social scenes). However, TD adults increased their attention to faces significantly more when watching two people communicate than did ASD adults, who increased their attention to a lesser degree. Further analysis revealed that ASD adults persisted in looking at hands and toys, even when observing two people communicate in a *rich* social scene.

**Conclusions:**

Diminished gaze to faces when observing two people communicating may lead to fewer opportunities for social learning and subsequent reductions in social knowledge. Naturalistic measures of contextual modulation could help identify areas of need for individuals learning about the social world and could become treatment targets to improve everyday social learning.

## Background

Individuals on the autism spectrum^1^ struggle to achieve success during everyday social interactions [4] and demonstrate atypical social learning [5]. Observing social interaction—or “social eavesdropping”—can lead to increased social knowledge [6–9] and may be a useful learning tool for people with social deficits. To learn from social observation, people must do at least two things: First, they must focus their attention on the most informative parts of the scene (e.g., the *face* is a good source of information about how someone is feeling, whereas the *hands* are useful to determine what someone is doing [10]). Second, they must be able to flexibly adapt their looking behaviors in real time; faces, hands, or objects may be more or less informative depending on context. Relative failures to adjust visual attention in response to contextual differences could lead to missed opportunities for social learning [11], with early inattention to faces (and over-attention to objects) leading to compounding downstream effects on developing social skills and cognition that could persist into adulthood [5]. In this study, we use an established eye-tracking paradigm to test whether and how autistic adults strategically adjust their gaze to capture key social information (faces) in scenes that are socially *rich* or socially *lean*.

### How do people decide where to look?

Real-world social scenarios contain a wide variety of potential visual targets, including people (faces, hands, bodies); animals; objects; and backgrounds. In most social situations, attending to faces is an informative strategy. Faces are afforded special status from birth [12], and face-to-face mutual looking shortly after birth can help infants establish caregiver-child attachments, thereby improving access to social spheres that provide food, warmth, and safety [13]. As children grow older, faces provide information about eye gaze direction (joint attention), which can enhance survival by providing information about threats or food opportunities in the environment [14]. Faces also produce language, and audiovisual synchrony between sounds and lip movements can help disambiguate important verbal signals in noisy environments [15]. Smiles or frowns shed light on the emotional status of the person producing the expression [16], and contiguity or contrast among internal facial features or facial features and body language can convey complex social messages (e.g., a disgusted facial expression combined with a relaxed posture means something different than the same expression combined with an angry posture [17]). Despite the enhanced status of faces for survival in a social group, however, faces are not always the most informative part of a scene.

### Is preferential attention to faces always the best strategy?

As people move through the world, they encounter situations where faces are more or less valuable sources of information. For example, attending to faces is very useful when observing two people actively coordinating their efforts to achieve a common goal. Facial expressions may indicate whether joint work is proceeding successfully; eye contact facilitates communication; and switching gaze between the partner and the task (shared attention) can indicate what aspect of the job to focus on next. On the other hand, the facial expressions of people engaging in two unrelated activities and not communicating with one another may be less valuable sources of information than their hands or adjacent objects. Reduced focus on eyes and faces is associated with ASD and generally considered detrimental [18], but the value of looking at faces is relative and varies by context.

### Social attention in ASD

Gaze to social stimuli has been extensively studied in autism [19–22], and it has been argued that early impairments in social attention have cascading downstream effects on later social skills and cognition [5]. In one of the first studies to use eye tracking to study visual attention in ASD, adults demonstrated less attention to the eyes of actors than TD controls, as well as more attention to mouths, bodies, and background objects [18]. This finding—that ASD individuals fixate relatively less on certain key social stimuli and more on non-social stimuli than TD controls—is consistent with clinical observations of reduced social attention in ASD [4] and has subsequently been replicated across a range of ages [11, 21, 23–27] but see [28–31].

### Gaze adaptation in ASD

At least two studies of younger children with ASD found that they struggle to flexibly adapt their eye gaze patterns to accommodate different social contexts [32, 33]. However, little is known about how ASD adults adapt their gaze behavior when viewing social scenes that contain more or less socially-rich communicative content. While it is possible that childhood impairments in the contextual modulation of social gaze persist throughout the lifespan in ASD, it is also possible that this ability emerges on an extended timeline in ASD, similar to skills in other domains that are “delayed but not deviant” [34–36]. Understanding how ASD adults allocate their visual resources in various social contexts could lead to personalized interventions aimed at enhancing attention to informative aspects of a scene, potentially leading to important gains in social learning from observation.

### The present study

In this study, we use eye tracking to test whether ASD adults and TD adults use social context to guide their gaze behavior. In contrast to studies that simply measure attention to social vs. nonsocial stimuli [21], our paradigm explores whether adults adjust their gaze patterns to efficiently capture social information when scenarios systematically incorporate more or less communicative content. Based on prior literature showing specific difficulty processing dynamic social stimuli in ASD [37, 38], we expected that ASD adults would demonstrate less looking toward faces overall than TD adults. We further hypothesized that the TD group would modify their gaze patterns when observing two people communicating, such that they significantly increase their gaze to faces in communicative contexts (*rich* social scenes) relative to non-communicative contexts (*lean* social scenes, which served as a baseline for social interest and activity monitoring, without social communication). Finally, we hypothesized that the ASD group would increase their gaze to faces in a communicative context as well, but to a significantly lesser extent (perhaps due to rigid perceptual processing patterns [39, 40]), instead of looking at hands or background objects.

## Methods

### Participants and clinical measures

ASD adults (*N* = 29) and TD adults (*N* = 29) were recruited through existing research and clinical databases at the Children’s Hospital of Philadelphia and the University of Pennsylvania, recruitment fliers, and social media posts. Participants provided written informed consent to undergo eye tracking as a part of a larger study (TUNE-In [41]) approved by the Institutional Review Boards of the Children’s Hospital of Philadelphia and the University of Pennsylvania. An expert doctoral-level clinician determined participants’ diagnostic status using DSM-5 criteria [4], informed by an assessment battery including the Autism Diagnostic Observation Schedule – 2nd Edition (ADOS-2 [42]). Two participants in the TD group were excluded due to insufficient gaze data (< 20% captured), and one participant in the ASD group was excluded due to missing intelligence quotient (IQ) information. No participants were excluded for failure to calibrate or calibration errors. Table 1 contains age; sex; race; IQ estimates (full-scale IQ was estimated using the WASI-II [43]); Broad Autism Phenotype Questionnaire self-report total scores (BAP-Q [44], higher scores indicate more symptoms of the broader autism phenotype); and ADOS-2 calibrated severity scores (CSS, range 0–10, higher scores indicate more severe autism symptoms [45, 46]) for the final sample (ASD = 28, TD = 27). Sample size was based on a power analysis conducted for the primary study, which was a pilot study of the efficacy of TUNE-In (a social communication intervention designed for adults on the autism spectrum (R34MH104407, PI, Brodkin). There was no stop rule for participant data collection; every participant in TUNE-In agreed to undergo eye tracking.

**Table 1:** Participant demographic and clinical characteristics, mean (SD). Group differences were tested using linear models (R function “lm”) for continuous variables and chi-square (*χ*^2^) with Yates’ continuity correction (R function “chisq.test”) for categorical variables

	ASD (*N* = 28)	TD (*N* = 27)	Difference
Age (years)	27.04 (7.39) Range 21–49	28.19 (9.11) Range 20–48	*t*(53)= .52 *p* = .61
Sex	25 male, 3 female	23 male, 4 female	*χ*^2^ = .003 *p* = .96
Race	African American/Black = 3 Asian/Pacific Islander = 3 White = 22	African American/Black = 3 Asian/Pacific Islander = 3 White = 20	*χ*^2^ = 1.14 *p* = .77
Full scale IQ	104 (20) Range 72–140	112 (10) Range 97–136	*t*(53) = 2.03 *p* = .05*
BAP-Q	3.64 (.70) Range 2.19–5.03	2.44 (.52) Range 1.47–3.47	*t*(53) = 7.25 *p* < .001**
ADOS-2 CSS	6.96 (2.05) Range 2–10	1.19 (1.10) Range 0–5	*t*(52) = 12.78, *p* < .001**

One TD participant did not report race, and one TD participant had missing ADOS-2 scores

*p < .05, **p < .01, ***p < .001

### Eye-tracking procedure

Participants were seated in a quiet room ~ 60 cm from a Tobii X120 infrared eye tracker, programmed to collect data at 60 Hz. Stimuli were presented using Tobii Studio and displayed on a 30-in. computer monitor that was placed on an adjustable table. Participant gaze was calibrated using a standard five-point Tobii calibration, which was repeated until eye gaze data were visible all 5 points. Calibration was not repeated during the 5.68-min task, and AOIs were drawn slightly outside the stimulus bounds to account for possible drift due to slouching and other participant movement (e.g., face AOIs were drawn as ovals that captured the actor’s face, part of her/his neck, and part of her/his hairline). After calibration, participants were instructed to watch a short movie while trying to remain still.

### Eye tracking paradigm

**Fig. 1:**
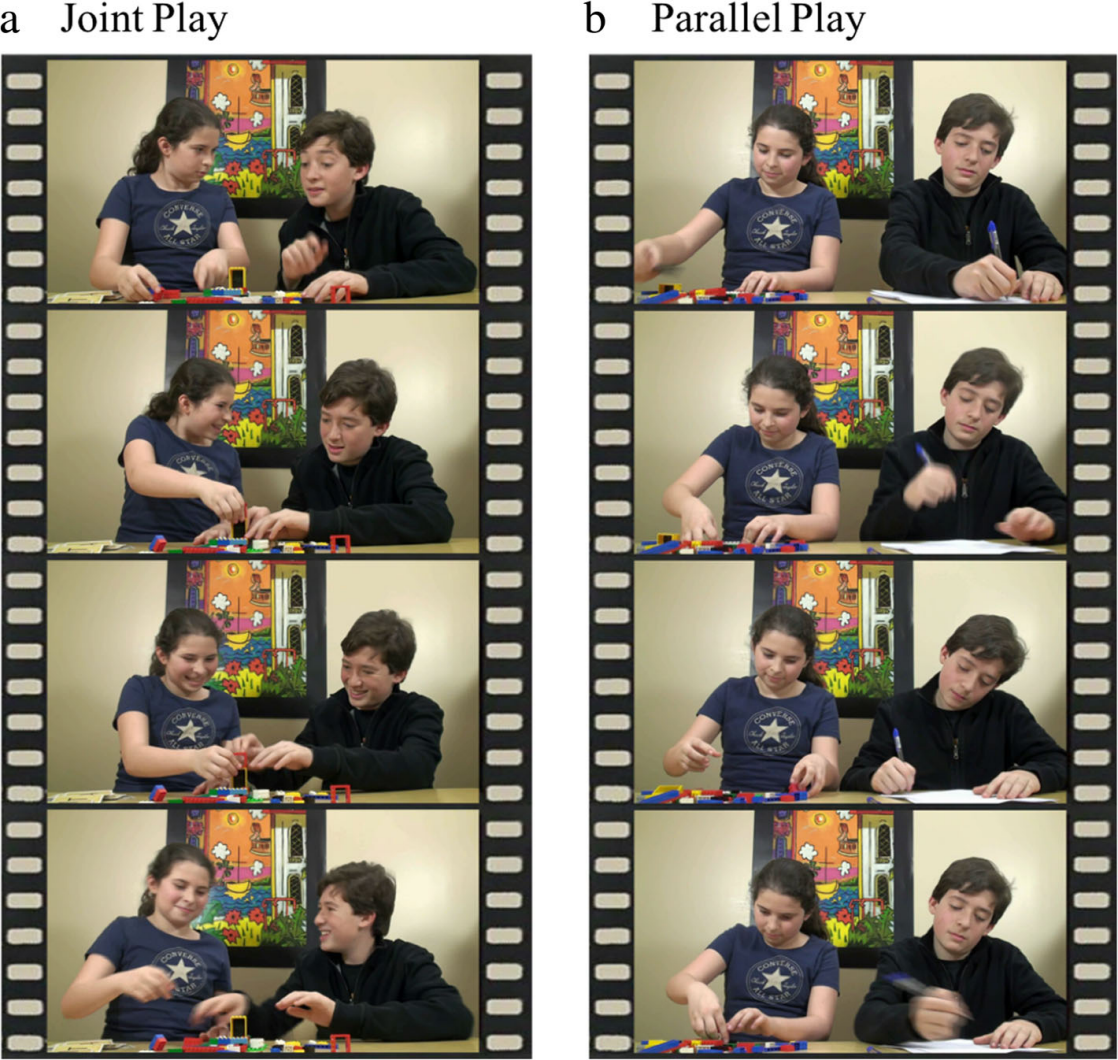
Examples of Joint Play (**a**) and Parallel Play (**b**) clips for a single sibling pair. Child actors and their parents provided permission to use these images

Participants watched a silent movie comprised of 22 sequential clips of 11 sibling pairs of school-aged children playing with toys at a table or on the floor. Children were filmed playing in various playrooms with objects (e.g., paintings, toys, light switches) visible in the background, and various toys (e.g., playing cards, paper and pencil, “barrel of monkeys” game) available for play on the table and floor. The videos were naturalistic; no specific guidelines were provided to child actors apart from instructions to use only nonverbal communication and to not look at the camera. Clips were taken from longer original recordings and were selected to maximize social salience (i.e., dyads were rated as appearing natural and spontaneous with positive affect). In the first Condition (Joint Play), two children actively played with one set of toys together (*rich* social scene), with another set of toys visible nearby. The children engaged in nonverbal communication that included eye contact and smiling (Fig. 1a). In the second Condition (Parallel Play), two children smiled and played with separate sets of toys, not engaging in any kind of communication with one another (*lean* social scene; Fig. 1b). Each condition included 11 trials (15.5 s each) separated by a 1-s white cross-hair in the center of a black background (total duration excluding cross-hairs = 341 s, 5.68 min). Trials were interspersed, with conditions shown in a fixed pseudorandomized order. For additional details on this task, please refer to [37], where the paradigm was described as the “Interactive Visual Exploration” task and not analyzed by condition; participants in that study and this one are non-overlapping.

### Eye-tracking parameters

Gaze data was exported from Tobii Studio using a filter based on the Velocity-Threshold Identification (I-VT) fixation classification algorithm [47]. Fixation parameters were as follows: Gap fill-in using linear interpolation was enabled, with a maximum gap length of 75 milliseconds. An average of the right and left eyes was used to calculate fixation. Noise reduction was disabled, and the velocity calculator was set at 20 ms. Adjacent fixations were merged, with the maximum time between merged fixations set to 75 ms and the maximum angle between merged fixations set to 0.5°. Merging fixations close in time and proximity prevents longer fixations from being separated into shorter fixations because of data loss or noise. Fixations shorter than 30 ms that did not meet the criteria for merging were discarded.

### Areas of interest (AOIs)

AOIs were manually drawn around faces (Face), hands playing with toys (HWT—often overlapped bodies), and Background Objects by the first author from [37]. Key frames were created using Tobii Studio and were adjusted by hand as needed to maintain the intended stimuli of interest (Face, Hands with Toys, or Background Objects) within the target AOI space at all times. Given the naturalistic character of the stimuli, AOIs were not the same size and shape across all scenes and changed size/shape in the course of a scene. The Background Object AOIs included peripheral toys that were not currently being played with (all scenes contained equal numbers of toys), wall art, light switches, etc. Videos were naturalistic, so the presence and nature of non-toy background objects were allowed to vary and did not differ systematically by condition. An AOI that covered the entire screen for each trial was also created, in order to measure overall visual attention (Fullscreen). AOIs of each type (Face, HWT, Background Objects, Fullscreen) were grouped within each scene for analysis. Fixation durations for each AOI group were summed across all scenes within each condition, to create Total Fixation Duration variables for each AOI type (in seconds).

Stimuli were shown on a screen with pixel (p) resolution 1920p × 1080p. Face AOIs were ovals approximately 340p wide (~ 9 cm) and 440p tall (~ 11.64 cm), depending on individual child actors, which translates to visual angles of ~ 8.58° by ~ 11.08° at 60-cm viewing distance. Key frames were used to adjust oval size and location as actors moved in 3D space (i.e., ovals became larger as actors moved closer to the camera and smaller as they receded), with dynamic interpolation between key frames. The size, shape (irregular polygons), and movement of AOIs for Hands with Toys were highly variable, with dimensions ~ 27° wide by ~ 14° tall and approximately 50 key frames per 11.5-s scene. Background objects varied in size, shape, and number in each scene. Objects were static in space, but AOIs were adjusted as necessary to account for actor movement (i.e., background AOIs only accounted for Background Objects while they were visible).

### Dependent variables and statistical approach

Analyses were conducted in R (primary package, lme4 [48]). Four linear mixed effects models tested predictors of Total Fixation Duration (sum of fixations in seconds) in each AOI group (Fullscreen, Face, HWT, Background Objects). AOI models for Face, HWT, and Background Objects included total attention the full screen and full-scale IQ as covariates, with diagnostic Group (TD = 0, ASD = 1), Condition (Parallel Play = 0, Joint Play = 1), and the interaction between Group and Condition as fixed effects, and participant ID as a random effect (to account for dependencies between observations).

### Preliminary analyses—attention

There was no significant interactive effect of Group and Condition on overall attention to the paradigm (estimate = − 2.69, SE = 3.44, *t* = −.78, *p* = .44; overall raw mean = 70%, SD = 14%, range 38–88%), nor was there a significant main effect of Group (estimate = 4.88, SE = 6.87, *t* = .71, *p* = .48). There was a significant main effect of Condition (estimate = 10.25, SE = 2.45, *t* = 4.18, *p* = .0001) such that all participants looked longer at the socially rich Joint Play Condition (mean = 74%, SD = 14%, range 38%–89%) than the socially lean Parallel Play Condition (mean = 69%, SD = 9%, range 32%–87%). Subsequent analyses included total gaze to the full screen as a covariate (see the “Dependent variables and statistical approach” section).

## Results

### Gaze to Faces

**Fig. 2:**
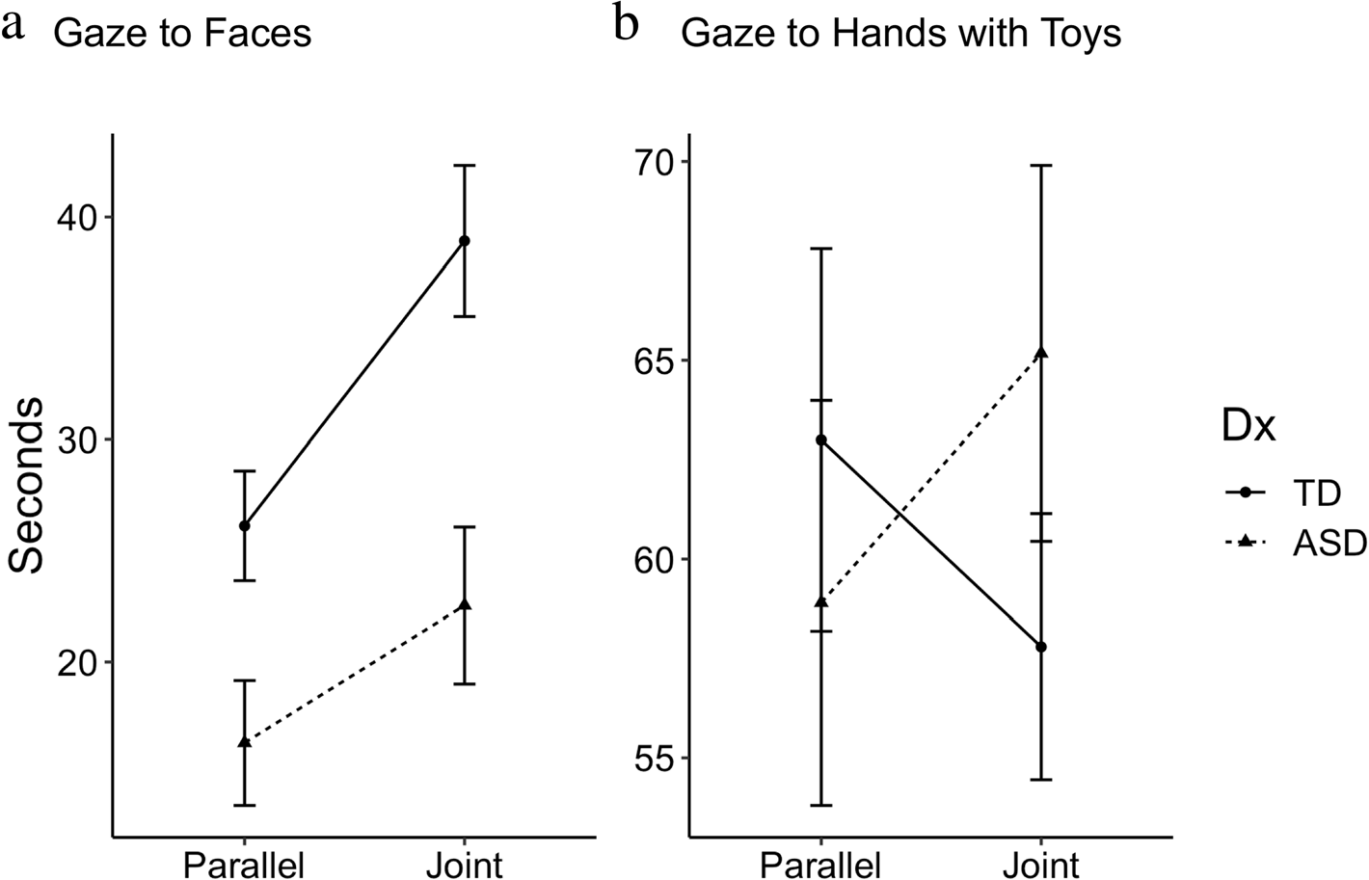
Gaze duration to Faces (**a**) and HWT (**b**) in the Joint and Parallel Play Conditions. Confidence intervals represent one standard deviation from the mean in either direction

To assess whether ASD and TD participants modulated their attention to Faces according to communicative context, we conducted a linear mixed effects model with Group (ASD, TD) and Condition (Joint Play, Parallel Play) and the interaction between Group and Condition as predictors of Total Fixation Duration. Results revealed a significant two-way interactive effect of Diagnosis and Condition on gaze duration to Faces (Table 2), suggesting significantly reduced contextual modulation to Faces by the ASD group in the socially rich Joint Play Condition (Fig. 2a), compared to the TD group. Significant main effects of Group (ASD < TD) and Condition (Joint Play > Parallel Play) on gaze to Faces emerged.

**Table 2:** Results of a linear mixed effects model predicting looking time to Face AOIs

	Estimate	Std. error	df	*t* value	*P* (> |*t*|)
(Intercept)	13.80	16.67	51.28	0.83	0.41
Att_fullscreen	0.07	0.05	51	1.50	0.14
FSIQ4.z	− 0.55	2.24	51	− 0.25	0.81
DxASD	− 10.09	4.52	59.12	− 2.23	0.03*
Cond_Joint	12.81	1.76	53	7.30	0.01^−8^***
DxASD:Cond_Joint	− 6.63	2.46	53	− 2.70	0.01**

*p < .05, **p < .01, ***p < .001

### Gaze to HWT and Background Objects

To determine whether gaze to other parts of the scene differed by group and social context, we tested two additional linear mixed effects models. The first revealed a significant interactive effect of Diagnosis and Condition on gaze to HWT (Estimate = 11.47, SE = 4.42, *t* value = 2.60, *p* = .01), suggesting that the ASD group looked more at Hands with Toys during the socially rich Joint Play Condition than the TD group (Fig. 2b). There were no main effects of Diagnosis or Condition on gaze duration to HWT. The second model revealed a significant main effect of Condition on gaze to Background Objects (Estimate = − 3.17, SE = 1.02, *t* value = − 3.11, *p* = .003), suggesting that all participants looked less at Background Objects during the Joint Condition. There was no significant main effect of Diagnosis on gaze to Background Objects and no significant interaction between Diagnosis and Condition. For all raw means and standard deviations, see Table 3.

**Table 3:** Raw means and standard deviations of Total Fixation Duration to each AOI type (in seconds)

Condition	Diagnosis	Face	Hands with Toys	Background Objects
Joint	ASD	21.96 (18.62)	64.24 (25.07)	5.10 (5.55)
	TD	38.93 (17.66)	57.80 (17.40)	3.43 (3.09)
Parallel	ASD	15.82 (14.87)	58.28 (26.65)	8.23 (6.38)
	TD	26.12 (12.84)	63.00 (24.98)	6.59 (6.29)

## Discussion

Complex, dynamic social scenes are the rule in daily life—not the exception. In this study, we tested the extent to which ASD adults and TD controls adjusted their gaze when observing scenes that were socially *lean* (two people interacting with toys, but not with each other) vs. socially *rich* (two people engaging in dynamic nonverbal communication with one another)*.* As predicted, we found that TD adults looked more at faces in the socially rich context of two people communicating, and less at faces when the people in a scene were not interacting with one another. Displaying a similar pattern, ASD adults modulated their attention to faces up in the Joint Play context and down in the Parallel Play context. However, a significant interactive effect of Condition and Diagnosis on gaze to Faces indicated that the *magnitude* of adjustment distinguished ASD from TD participants. Thus, the TD group modulated their attention to faces to a greater extent than the ASD group in the Joint Play Condition, who instead looked more at people’s hands and toys. This pattern of results could have two potential causes, both of which are plausible in light of prior studies. First, the ASD group may have been slower or less willing to adjust to contextual differences in video stimuli [11]. This would explain why they persisted in looking at hands and toys in the Joint Play Condition (both groups preferred looking at hands and toys overall, but especially in the Parallel Play Condition) and is consistent with reports of reduced perceptual and behavioral flexibility in this population [49], as well as a preference for looking at objects [39, 40]. Alternatively, the TD group could have looked relatively more at faces than the ASD group overall, and specifically during the socially rich Joint Play Condition, due to powerful instinctive biases toward attending to communicative stimuli that are diminished in autistic adults (the Social Motivation explanation [5, 50]). We expect that both of these explanations partially underlie the pattern of results reported here.

Consistent with prior research showing overall reduced attention to social stimuli in ASD adults [51], we found a main effect of diagnostic status on gaze to faces, such that autistic adults watching dynamic, naturalistic social scenes looked less at faces than TD controls in both conditions. Combined with results suggesting reduced contextual modulation in response to social stimuli, our study suggests a complex set of social observation impairments in ASD that include [1] overall reductions in social attention and [2] difficulty adjusting observation patterns in response to different scenarios. These two challenges likely map to distinct underlying mechanisms that will respond to different intervention approaches; while the first could be treated by targeting social motivation and reward processes, the second will likely respond to interventions that address flexibility and attention switching.

Of note, all participants in this study spent the most time gazing at Hands with Toys, regardless of condition (Table 3). Given that activity monitoring is an important way to learn [22] and watching a person’s hands precipitates foundational social behaviors including joint attention [51], it was especially critical to include a baseline comparison condition—Parallel Play (children interacting with toys separately, without communicating with one another) in this study. With this condition as a comparison, we were able to pinpoint the effect of adding social communication on gaze behavior to faces and activities in TD adults—and then compare those patterns to ASD adults. One potential takeaway from the current study is that when social communication between two people occurs, typical adults adjust their distribution of attention to capture that important stream of information and in doing so, they necessarily downregulate their gaze to different parts of the scene. Even then, TD adults do not cease activity monitoring altogether; as noted, all participants attended to Hands with Toys the most in both conditions. Our results simply suggest that while adjustments to capture communicative information from faces occurs in both TD adults and ASD adults, there is significantly less adjustment in the clinical group.

This study adds to the literature in several important ways. First, a recent meta-analysis of eye tracking literature in ASD concluded that the richness of social content (including the number of persons depicted) determines whether or not diagnostic group differences are found [24]. In contrast to much prior work, but consistent with Klin et al., the current study used dynamic stimuli that included two people, rather than just one. This may have made the task more difficult for individuals with social challenges and enhanced diagnostic group differences. We also extend our prior work in children [37] by demonstrating that adults with ASD respond atypically to stimuli of varying social richness—suggesting persistent deviance in this area of social perception, rather than delay. Second, unlike a recent study assessing contextual modulation of gaze [32], actors in our video did not address the participant directly; rather, they communicated with one another. This is akin to observing two people interacting during a meeting, at a coffee shop, or on a playground, and is consistent with the approach taken by Klin et al. and Lonnqvist et al. [52]. Finally, rather than simply measuring attention to social vs. nonsocial stimuli [21], our paradigm was designed to capture the *magnitude of difference* when observers modify their gaze patterns to capture relevant information from different kinds of social scenes that do or do not contain communicative content, revealing a smaller magnitude of adjustment in ASD. Taken together, these differences mark our study as an independent contribution to the field’s understanding of social observation in adults on the autism spectrum.

### Limitations and future directions

This study has a number of limitations. Although we employed dynamic and naturalistic video stimuli, our method is still screen-based and thus faces a generalizability/ecological validity problem that would be solved by measuring gaze during real-world observation of social interactions. Although a handful of studies exist [30, 53, 54], further research using head mounted or glasses-based eye tracking is needed to clarify how social observation works in the real world (e.g., how do individuals with ASD observe colleagues during work meetings or learn from watching peers on the playground?). The use of child actors for measuring social attention in adult participants may seem unusual on its face; however, research suggests that adults—whether or not they are parents—have a tendency to preferentially attend to images of infants and children vs. teens and other adults [55], making child actors a logical choice for testing adult patterns of social observation. Future studies using adult actors are warranted.

Our sample did not have a sufficient number of autistic women to test whether contextual modulation differs by sex; given emerging research suggesting that social attention may be greater in autistic females compared to males (Harrop et al., under review), this is a promising future research direction. This study did not include adults with co-occurring intellectual disability, although we did have a wide IQ range in the ASD group. However, our findings suggest that studying autistic adults without co-occurring intellectual disability continues to be of value, since this group is still challenged to effectively allocate visual attention in temporally dynamic contexts and could benefit from targeted interventions to improve this skill set. We did not have a granular measure of behavioral flexibility in our clinical battery; future eye-tracking research should test whether executive dysfunction is an underlying mechanism that leads to contextual modulation difficulties for autistic adults, as it does in other modalities [49]. In contrast to some prior work, the current study examined face looking as a whole and did not separate out internal facial features such as eye and mouth areas. This approach is consistent with our primary goal, which was to examine the overall distribution of visual attention to social and non-social aspects of scenes that varied in social richness, rather than examining how participants parse individual facial features.

## Conclusions

Autistic adults struggle with everyday social interaction, and differences in how they look at the world may make it harder to learn from observing others. In this study, we used eye-tracking technology to measure the way autistic adults watch others interact socially. We found that autistic adults adjust their visual attention to faces in communicative contexts to a lesser extent than TD adults, instead of looking more at hands and objects. Thus, adults on the autism spectrum may miss key information during “social eavesdropping” when they fail to fully modulate their visual attention to the faces of two people communicating. These findings lay the groundwork for future treatment efforts aimed at improving social learning and add to the literature documenting nuanced differences in social attention across the lifespan in ASD.

## Abbreviations

ADOS-2: Autism Diagnostic Observation Schedule – 2nd Edition
AOI: Area of interest
ASD: Autism spectrum disorder
CSS: Calibrated severity score
HWT: Hands with Toys
TD: Typically developing

## Footnotes

1 N.B. In this paper, we refer to individuals diagnosed with ASD as “autistic adults,” “adults on the autism spectrum,” or “ASD adults.” Many self-advocates, including the leaders of the Autistic Self Advocacy Network, prefer the term “autistic” over “person with autism” [1–3]; we honor that decision.
